# Risk for human tick-borne encephalitis, borrelioses, and double infection in the pre-Ural region of Russia.

**DOI:** 10.3201/eid0703.010319

**Published:** 2001

**Authors:** E I Korenberg, L Y Gorban, Y V Kovalevskii, V I Frizen, A S Karavanov

**Affiliations:** Gamaleya Research Institute for Epidemiology and Microbiology, Russian Academy of Medical Sciences, Moscow, Russia.

## Abstract

We assessed the risk for human tick-borne encephalitis (TBE), ixodid tick-borne borrelioses, and double infection from 1994 to 1998 in Perm, which has among the highest rates of reported cases in Russia. We studied 3,473 unfed adult Ixodes persulcatus ticks collected from vegetation in natural foci and 62,816 ticks removed from humans. TBE virus and Borrelia may coexist in ticks.


### Risk for Human Tick-Borne Encephalitis, Borrelioses, and Double Infection in the Pre-Ural Region of Russia

**Edward I. Korenberg,* Lidiya Ya. Gorban',† Yurii V. Kovalevskii,* Vladimir I. Frizen,† and Andrei S. Karavanov‡**
*Gamaleya Research Institute for Epidemiology and Microbiology, Russian Academy of Medical Sciences, Moscow, Russia; †Perm Center of State Sanitary-Epidemiologic Inspection, Perm, Russia; and ‡Chumakov Institute of Poliomyelitis and Viral Encephalites, Russian Academy of Medical Sciences, Moscow, Russia

We assessed the risk for human tick-borne encephalitis (TBE), ixodid tick-borne borrelioses, and double infection from 1994 to 1998 in Perm, which has among the highest rates of reported cases in Russia. We studied 3,473 unfed adult *Ixodes persulcatus* ticks collected from vegetation in natural foci and 62,816 ticks removed from humans. TBE virus and *Borrelia* may coexist in ticks.

Tick-borne encephalitis (TBE) and infections of the Lyme borreliosis group, or ixodid tick-borne borrelioses (ITBB) (1), are widespread in Russia. In 1996, 1997, and 1998, cases of these diseases totaled approximately 16,650, 11,350, and 14,700, respectively. Most cases were acquired from the bite of an adult *Ixodes persulcatus* tick. In some regions west of the Volga River, the *I. ricinus* tick may also transmit these infections. The geographic distribution and epidemiology of TBE and ITBB in Eurasia are almost identical (2).

These data suggest that double infection by TBE virus and *Borrelia* may result from transmission of both pathogens from double-infected ticks to humans (2). Such ticks are present in natural foci, and the occurrence of TBE virus and *Borrelia* is independent in ticks rather than mutually exclusive. The prevalence of *Borrelia* in unfed *I. persulcatus* ticks with and without TBE virus is virtually identical, and the same is true for TBE virus prevalence in ticks with and without *Borrelia* infection. Concentrations of virus and *Borrelia* in double-infected ticks are not correlated. In the natural mixed foci of TBE and ITBB, interannual changes in the prevalence of virus and spirochetes in ticks are virtually parallel. The coexistence of these microorganisms in their principal vectors, which promotes simultaneous infection in humans bitten by ticks, is apparently an important precondition for the relative autonomy of conjugate parasitic systems formed by TBE virus and *Borrelia* (3). However, prevalence of both pathogens in ticks collected from vegetation has not previously been compared with their prevalence in ticks removed from humans.

Cases of double infection were identified for the first time during 1986 to 1988 in Austria and northwestern European Russia (4,5). They have been described in the species range of the *I. ricinus* tick (6) but occur more frequently in areas where *I. persulcatus* ticks are abundant (7,8). We describe long-term studies assessing the risk for human double infection compared with that for separate TBE and ITBB infection in a region with a consistently high level of both diseases (1).

Address for correspondence: E.I. Korenberg, Gamaleya Research Institute for Epidemiology and Microbiology, Gamaleya Str. 18, Moscow, 123098 Russia; fax: 7-095-115-1255; e-mail: focus@edkor.msk.ru

### The Study

The study was performed from 1994 to 1998 in the city of Perm, located in the Pre-Ural region, which has among the highest rates of reported cases in Russia. In this area, the *I. persulcatus* tick is the only vector transmitting these infections to humans (1,2,9,10). Only two *Borrelia* species (*B. garinii* and *B. afzelii*) circulate in this area, but the frequency of *B. garinii* in ticks, rodents, and patients is substantially higher (11). Each year, unfed adult ticks were collected from vegetation in several areas where people are exposed to ticks. In most cases (>98%), people are bitten by adult *I. persulcatus* ticks, and only rarely by nymphs (12). Adult ticks were removed from patients who sought medical treatment at a city laboratory designated for the diagnosis and prophylaxis of TBE and ITBB. We studied 66,289 adult *I. persulcatus* ticks, including 3,473 collected from vegetation and 62,816 removed from humans. Some ticks remained attached 1 to 6 days before being removed, but in most cases (~90%) people came to the laboratory <1 day after the tick attached (12), virtually before it began to feed.

Only living ticks were studied for *Borrelia* infection by dark-field microscopic analysis of tick gut contents in standard vital preparations at a magnification of X600. Two hundred fifty microscopic fields per preparation were studied, in 1,797 unfed ticks collected from vegetation and 7,442 ticks removed from patients. For virologic analysis, 1,676 unfed ticks were individually homogenized in 1 mL of medium 199 with Earle's salts (Sigma M3769, St. Louis, MO) containing 25% inactivated normal calf serum. Virus was isolated from the supernatant in cultures of an established pig kidney cell line and identified by the direct fluorescent antibody method and in hemagglutination inhibition and complement fixation tests with specific antibodies from ascitic fluid (13). In addition, all ticks removed from humans were studied on the same day to determine TBE virus by indirect immunofluorescent assay

(14); 7,442 of these ticks were concurrently analyzed for infection with *Borrelia*.

Both TBE and ITBB are reportable diseases for official statistics throughout Russia. Diagnosis is established on the basis of clinical signs and serologic evidence. We used these data for the total population of Perm (slightly over 1 million) on the number of TBE and ITBB cases and requests for medical aid to remove attached ticks.

Calculated percentages were analyzed by using a confidence interval based on double sampling error ($\pm 2m_p$). Significance of differences between mean values ($p<0.05$) was assessed by the Student *t* test. The correlation coefficient r was determined by Kendall rank correlation analysis. The degree of independence of phenomena under study was estimated by 2x2 contingency analysis for calculating chi square at $p<0.05$ and df = 1.

*Borrelia* prevalence in *I. persulcatus* ticks removed from humans varied from 25% to 35% in different seasons (Table 1). A 5-year series of data on the proportion of *Borrelia*-infected ticks from vegetation in natural foci compared with those removed from people showed good correlation (r = 0.71), indicating that changes in prevalence occurred in parallel.

Table 1. Prevalence of infection by *Borrelia burgdorferi* sensu lato (%) in unfed adult *Ixodes persulcatus* ticks collected from vegetation and removed from humans

| | Ticks collected from vegetation | | Ticks removed from humans | | |
|---|---|---|---|---|---|
| Year | No. | Prevalence of infection($p \pm 2m_p$) | No. | Prevalence of infection ($p \pm 2m_p$) | *t* |
| 1994 | 220 | 31.3 ± 6.2 | 878 | 27.3 ± 3.0 | 1.2 |
| 1995 | 232 | 34.9 ± 6.3 | 833 | 25.2 ± 3.0 | 2.7 |
| 1996 | 500 | 46.8 ± 4.5 | 2,388 | 35.5 ± 2.0 | 4.5 |
| 1997 | 451 | 25.0 ± 5.7 | 1,535 | 27.9 ± 2.3 | 0.9 |
| 1998 | 394 | 27.4 ± 4.5 | 1,808 | 28.9 ± 2.1 | 0.6 |
| Total | 1,797 | 35.1 ± 2.2 | 7,442 | 30.0 ± 1.1 | 4.1 |

$\pm 2m_p$ = confidence interval based on double sampling error.

Pairwise comparison for each year (1994, 1997, and 1998) of *Borrelia* prevalence in ticks removed from patients and collected from vegetation demonstrated that they were almost similar (t<2), whereas in 1995 and 1996, as well as over the whole study period, the proportions of specimens with *Borrelia* among ticks that attached to people were slightly lower than in nature.

As in the case of *Borrelia*, the lowest incidence of specimens with TBE antigen among ticks removed from patients was recorded in 1995, and the highest was observed in 1996 (Table 2). As the virologic analysis of ticks collected from vegetation and removed from patients was performed by different methods, direct pairwise comparison, as in the case of *Borrelia* infection (Table 1), would not be valid. However, in any year, the proportion of infected ticks from humans did not exceed that in ticks collected in nature (Table 2).

The degree of independence of TBE virus and *Borrelia* prevalence in concurrently studied ticks removed from humans was estimated by 2x2 contingency analysis. The resulting chi-square values, ranging from 0.22 to 1.01, provided evidence for lack of any dependence between the prevalence of TBE virus and *Borrelia* in ticks.

Table 2. Prevalence of infection by tick-borne encephalitis virus (%) in unfed adult *Ixodes persulcatus* ticks collected from vegetation and removed from humans

| | Ticks collected from vegetation | | Ticks removed from humans | |
|---|---|---|---|---|
| Year | No. | Prevalence of infection($p \pm 2m_p$) | No. | Prevalence of infection($p \pm 2m_p$) |
| 1994 | 664 | 21.5 ± 3.2 | 9,815 | 9.5 ± 0.6 |
| 1995 | 265 | 10.9 ± 3.8 | 8,523 | 5.5 ± 0.5 |
| 1996 | 167 | 35.3 ± 7.4 | 17,905 | 11.0 ± 0.5 |
| 1997 | 336 | 38.7 ± 5.3 | 12,443 | 8.8 ± 0.5 |
| 1998 | 244 | 15.2 ± 4.6 | 14,129 | 8.4 ± 0.5 |
| Total | 1,676 | 23.7 ± 2.1 | 62,816 | 9.0 ± 0.2 |

$\pm 2m_p$ = confidence interval based on double sampling error; Coefficient of correlation was r = 0.7.

Among ticks removed from patients, proportions with double infection from 1994 to 1998 were as follows: 2.2 ±1.0%, 1.2 ±0.7%, 3.3 ±0.7%, 2.3 ±0.8%, and 2.2 ±0.7%, respectively. In general, annual changes in TBE, ITBB, and double infection illness occur in parallel (Figure). Coefficients of correlation (r) between prevalence of these infections are as follows: TBE and ITBB, r = 0.73; ITBB and double infection, r = 0.53; TBE and double infection, r = 0.94; and TBE + ITBB and double infection, r = 0.84.

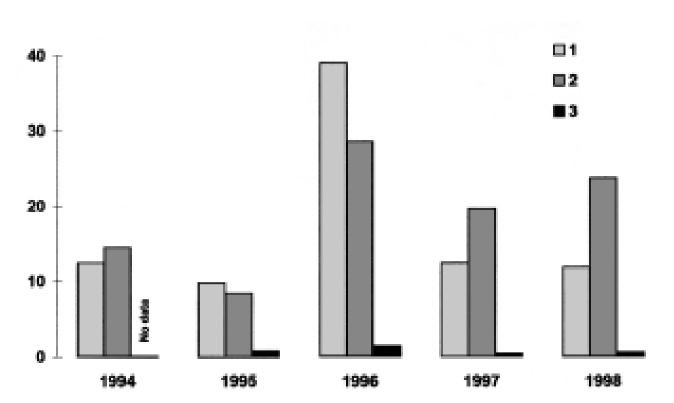

Figure. Rates of disease in Perm, Russia (per 100,000 residents), of 1) tick-borne encephalitis (TBE), 2) Ixodid tick-borne borreliosis (ITBB), and 3) double infection.

Based on the number of reported requests for medical treatment after tick bites and the pathogen prevalences in ticks, the probable frequency of contact with infected ticks was estimated (Table 3). These estimates, characterizing only the study group, substantially exceed the actual prevalence of illness for the population of Perm (Figure).

### Conclusions

Our data provide evidence for a distinct correlation between prevalence of TBE virus and *Borrelia* in *I. persulcatus* ticks collected from vegetation in natural foci and removed from humans. However, we found no evidence that ticks infected by TBE virus attach to people more often than uninfected ticks (15). *Borrelia* prevalence in ticks collected from vegetation and from patients is virtually identical. A trend toward increasing prevalence of spirochetes in unfed ticks in some years may be explained by technical difficulties in visualizing *Borrelia* under a microscope, which increase with engorgement (16).

Table 3. Frequency of possible contacts of people with ticks infected by pathogens of tick-borne encephalitis, ixodid tick-borne borrelioses, and both (double infection)

| Year | No. seeking medical treatment after tick bite | No. who had probable contact with ticks infected with: | | |
|---|---|---|---|---|
| | | TBE[a] | ITBB | Both (double infection) |
| 1994 | 1,260 | 120 | 345 | 27 |
| 1995 | 970 | 55 | 240 | 11 |
| 1996 | 2,070 | 230 | 735 | 68 |
| 1997 | 1,340 | 110 | 370 | 30 |
| 1998 | 1,280 | 115 | 370 | 28 |

[a]TBE = tick-borne encephalitis; ITBB = ixodid tick-borne borreliosis.

The results of our microbiologic and virologic testing of individual ticks removed from patients, as well as those of unfed *I. persulcatus* ticks from a natural focus (3), showed that the prevalences of their infection by TBE virus and *Borrelia* are independent. Our data do not support the concept that, in double-infected ticks, *Borrelia* circulation restricts TBE virus circulation; at the same time, the presence of virus in ticks may even promote *Borrelia* transmission (15,17,18). In fact, in double-infected *I. persulcatus* ticks, *Borrelia* and TBE virus do not interfere with one another.

This is why the prevalences of TBE, ITBB, and double infection illness correlate well, and human cases of double infection are more frequent in the regions where the incidence of both TBE and borrelioses is especially high. Hence, the hypothesis that TBE is replaced by borreliosis in conjugate foci (18) appears to be erroneous.

Knowing the frequency of human contact with ticks and the prevalence of both TBE virus and *Borrelia* in ticks, the probable annual frequency of combined exposure to these pathogens may be calculated for residents of a certain area. Our data show, however, that such calculations substantially exceed the current level of illness and the actual risk for acquiring not only double infection, but also each infection separately. This discrepancy results because not all bites from infected ticks transmit a dose of pathogen sufficient to cause clinical illness. In TBE, for example, most bites result in asymptomatic infection and immune response, and the mean ratio of clinically manifested to asymptomatic cases is 1:60 (19). However, this ratio varies widely by regions and year. Because most people remove ticks promptly (within <1 day [12]), a physiologic phenomenon called reactivation, which is required for some microorganisms before infectivity is attained, does not have time to develop. The risk for clinical illness of TBE and borreliosis is largely determined by highly infected ticks (20), which always account for only a few of all infected ticks in a population (21). In addition, among *I. persulcatus* ticks infected by *B. garinii* and *B. afzelii*, no more than half contain spirochetes in the salivary glands after a blood meal and are capable of transmitting them to bitten people (16). At the same time, only a fraction of bitten people seek medical help in removing an attached tick, and the actual number of people bitten by ticks is many times greater than reported (1). Therefore, prevalences of risk for human TBE, ITBB, and double infection may be no more than estimates. Nevertheless, the epidemiologic and clinical aspects of widespread tick-borne coinfections merit close attention from researchers and clinicians.

This study was supported in part by the Russian Foundation for Fundamental Research, grant no. 98-04-48127a.

Prof. Korenberg is chief of Laboratory of Vectors of Infections, Gamaleya Research Institute for Epidemiology and Microbiology, Russian Academy of Medical Sciences. He is also head of the Centre on Borrelioses for Russia and chairman of the committee of the Russian Academy of Medical Sciences on human infections with natural focality. His research interests include epidemiology and epizootiology of zoonoses, especially tick-borne infections.

### References

1. Korenberg EI. Ixodid tick-borne borrelioses (ITBBs), infections of the Lyme borreliosis group, in Russia: country report. In: Report of WHO Workshop on Lyme Borreliosis Diagnosis and Surveillance. Warsaw: Sanitati; 1995. p. 128-36.
2. Korenberg EI. Comparative ecology and epidemiology of Lyme disease and tick-borne encephalitis in the former Soviet Union. Parasitol Today 1994;4:157-60.
3. Korenberg EI, Kovalevskii YuV, Karavanov AS, Moskvitina GG. Mixed infection by tick-borne encephalitis virus and *Borrelia* in ticks. Med Vet Entomol 1999;13:204-8.
4. Kristoferitsch W, Stanek G, Kunz Ch. Doppelinfection mit Fruhsommermeningoenzephalitis- (FSME-) und *Borrelia burgdorferi*. Dtsch Med Wochenscrift 1986;111:861-4.
5. Mebel' BD, Beitrishvili GA, Zhivich MB, Osetrov BA, Kuznetsova RI, Korenberg EI, et al. Clinical progression of Lyme disease in the acute period. Med Parazitol Parazit Bolezni 1988;3:30-3 (in Russian).
6. Cimperman J, Maraspin V, Lotricfurlan S, Ruzicsabljic E, Avsic-Zupanc T, Picken RN, et al. Concomitant infection with tick-borne encephalitis virus and *Borrelia burgdorferi* sensu lato in patients with acute meningitis or meningoencephalitis. Infection 1998;26:160-4.
7. Korenberg EI, Kuznetsova RI, Kovalevskii YuV, Vasilenko ZE, Mebel' BD. Basic epidemiological features of Lyme disease in the northwestern Soviet Union. Med Parazitol Parazit Bolezni 1991;3:14-7 (in Russian).
8. Laikovskaya EE, Lesnyak OM, Volkova LI, Turova EL, Sokolova ZI, Khodyrev VN. Mixed infection by Lyme borreliosis and tick-borne encephalitis agents. In: Korenberg EI, editor. Problems of tick-borne borrelioses. Moscow; 1993. p. 93-8 (in Russian).
9. Ustinova OYu, Volegova GM, Devyatkov MYu, Gusmanova AG. Clinical-epidemiological features of tick-borne encephalitis in the Perm region. Zh Microbiol Epidemiol Immunobiol 1997;3:33-6 (in Russian).
10. Vorobyeva NN. Clinical picture, treatment and prevention of ixodid tick-borne borrelioses. Perm; 1998. p. 1-132 (in Russian).
11. Postic D, Korenberg E, Gorelova N, Kovalevskii Yu, Bellenger E, Baranton G. *Borrelia burgdorferi* sensu lato in Russia and neighbouring countries: high incidence of mixed isolates. Res Microbiol 1997;148:691-702.
12. Korenberg EI, Moskvitina GG, Vorobyeva NN. Prevention of human borreliosis after infected tick's bite. In: Cevenini R, Sambri V, La Placa M, editors. Advances in Lyme borreliosis research. Proceedings of the VI International Conference on Lyme Borreliosis. Bologna: Esculapio; 1994. p. 209-11.
13. Korenberg EI, Horakova M, Kovalevskii YuV, Hubalek Z, Karavanov AS. Probability models of the rate of infection with tick-borne encephalitis virus in *Ixodes persulcatus* ticks. Folia Parasitol 1992;39:85-92.
14. Matveev LE, Karavanov AS, Rubin SG, Semashko IV, Tsekhanovskaya NA, Presman EK. Comparative estimation of two immunofermantal test systems for determining antibodies to TBE virus. Vopr Virusol 1989;4:488-491 (in Russian).
15. Alekseev AN. Symbiotic relationships in the complex vector-pathogen system. Dokl Akad Nauk SSSR 1994;338:259-61 (in Russian).
16. Korenberg EI, Moskvitina GG. Interrelationships between different Borrelia genospecies and their principal vectors. J Vector Ecol 1996;21:178-85.

17. Alekseev AN, Burenkova LA, Vasil'eva IS, Dubinina EV, Chunikhin SP. The functioning of the foci of mixed tick-borne infections on the Russian territory. Med Parazitol Parazit Bolezni 1996;4:9-16 (in Russian).
18. Alekseev AN, Dubinina EV. Epidemiological aspects of mixed (encephalitis and borreliosis) tick-borne infections. Zh Infekts Patol 1996;3:5-9 (in Russian).
19. L'vov DK, Pokrovsky VI. Encephalites. In: Pokrovsky VI, editor. A manual of zoonoses. Leningrad: Meditsina; 1983. p. 7-10 (in Russian).
20. Korenberg EI, Kovalevskii YuV. Main features of tick-borne encephalitis eco-epidemiology in Russia. Zentralbl Bacteriol 1999;289:525-39.
21. Korenberg EI, Kovalevskii YuV. Variation in parameters affecting risk for human disease due to TBE virus. Folia Parasitol 1995;42:307-12.
